# The Cost and Intermediate Cost-Effectiveness of a Randomized Control Trial Delivering U=U Messaging to Increase HIV Testing Among South African Men

**DOI:** 10.9745/GHSP-D-24-00143

**Published:** 2026-08-10

**Authors:** P.J. Smith, D. Joseph Davey, L. Schmucker, L.-G. Bekker, A. Medina-Marino, H. Thirumurthy, A.M. Buttenheim, G. Meyer-Rath

**Affiliations:** a University of Cape Town, Desmond Tutu HIV Centre, Department of Medicine, Cape Town, South Africa.; b University of California Los Angeles, Division of Infectious Diseases, Geffen School of Medicine, Los Angeles, CA, USA.; c University of Cape Town, Division of Epidemiology and Biostatistics, School of Public Health and Family Medicine, Cape Town, South Africa.; d University of Pennsylvania, Center for Health Incentives and Behavioral Economics, Medical Ethics and Health Policy, Perelman School of Medicine, Philadelphia, PA, USA.; e University of Cape Town, Division of Men’s Health, Desmond Tutu HIV Centre, Cape Town, South Africa.; f University of Pennsylvania, Department of Psychiatry, Perelman School of Medicine, Philadelphia, PA, USA.; g University of Pennsylvania, Department of Family and Community Health, School of Nursing, Philadelphia, PA, USA.; h University of the Witwatersrand, Health Economics and Epidemiology Research Office (HE2RO), Faculty of Health Sciences, School of Clinical Medicine, Johannesburg, South Africa.; i Boston University, Department of Global Health, School of Public Health, Boston, MA, USA.; j Stellenbosch University, DSI-NRF Centre of Excellence in Epidemiological Modelling and Analysis (SACEMA), Stellenbosch, South Africa.

## Abstract

**Background:**

South African men have lower HIV testing uptake than their female counterparts, leading to lower antiretroviral treatment (ART) uptake and higher HIV-related mortality. A recent cluster-randomized trial in a high HIV prevalence area in Cape Town showed that relaying the “undetectable equals untransmittable” (U=U) message to South African men increased their HIV testing uptake compared to issuing standard testing invitations. We conducted an economic analysis based on this trial to inform decision-making regarding implementing U=U messaging in routine public-sector HIV care.

**Methods:**

We analyzed the mean cost of the U=U intervention and its intermediate cost-effectiveness over the control arm. Costs were estimated from trial expenditure data in 2020 South African rand (ZAR), converted using March 2020 US$ exchange rates. Shared trial costs were allocated to each arm based on participant volume or days of operation; capital costs, including the creation of the U=U message via human-centered design workshops, were annualized over 8 years. In a second scenario, trial cost was adjusted for potential implementation in routine care.

**Results:**

Peer promoters delivered 504 U=U invitations to test for HIV over 7 intervention days and 544 standard invitations over 5 control days in March 2020. Compared with standard invitations, men who received U=U messaging had 60% increased odds of getting tested for HIV. The average trial cost was $5.40 per U=U message delivered ($2.35 after adjustment for routine implementation), $388.47 per person testing HIV-positive ($168.99 adjusted), and $906.44 per person initiating ART ($394.31 adjusted). Within trial costs, staff, overhead, and research costs were the largest allocations, with each accounting for 20% to 30% of total costs. After routine implementation adjustment, the costs of message creation via human-centered design workshops, personnel (peer promoters, research staff), and supplies were the largest allocations. Cost of U=U messaging per person confirmed HIV-positive and initiating ART was lower than conventional HIV testing messaging and at the lower end of cost ranges of HIV self-test distribution models targeted at men ($62 to $7,936 per person confirmed positive and $117 to $8,198 per person initiating ART) established in previous work.

**Conclusion:**

Delivering tailored U=U messaging can increase HIV testing and ART uptake among men while saving costs over standard untargeted HIV testing messaging and other HIV self-test distribution models.

## Abstract (precis)

Tailored U=U (undetectable equals untransmittable) messages for South African men increased uptake of HIV testing and antiretroviral treatement and were more cost-effective than standard messages and other HIV testing models aimed at men.

## Teaser

**Key Findings:**

- This study analyzed the cost and intermediate cost-effectiveness of delivering “undetectable equals untransmittable” (U=U) messaging in community-based settings, focusing on its impact on men’s HIV testing, positive diagnoses, and linkage to care.
- Peer promoters delivered 504 U=U invitations over 7 intervention days and 544 standard invitations over 5 control days. Men who received U=U messaging had 60% higher odds of getting tested for HIV compared with those who received standard invitations.
- The cost per person confirmed HIV-positive and initiating antiretroviral treatment with U=U messaging was lower than conventional HIV testing invitations, and it was at the lower end of cost ranges for HIV self-test distribution models targeted at men from previous studies.

**Key Implication:**

- These findings demonstrate that human-centered design interventions, such as tailored U=U messaging, may be used to improve health behavior and reduce costs of increasing HIV testing uptake.

## INTRODUCTION

South African males are less likely than their female counterparts to know their HIV status, to test for HIV (85% versus 92%, respectively), to initiate antiretroviral treatment (ART) (67% versus 72%, respectively), and to reach viral suppression (82% versus 90%, respectively).^1^^–^^7^ Although the country’s ART scale-up since 2004 is associated with an increase in population life expectancy in South Africa, these disparities lead to a lower comparative survival prior to and while on treatment^3^^,^^8^ for males. To address this gap and to reach the UNAIDS target to test 95% of people living with HIV (PLHIV),^9^ men from settings with a high disease burden require tailored demand-creation messaging and services to improve their HIV testing uptake and continuation to viral suppression.^10^^–^^13^

While men are willing to engage with HIV services, there are a number of barriers preventing their access to conventional, facility-based HIV services,^10^ including that care is largely focused on women, generic information is often targeted toward women, long commutes, long waiting times, and a lack of confidentiality.^4^^,^^10^^,^^11^ Additionally, there is the potential for distress and stigma resulting from an HIV-positive diagnosis, which can cause men to avoid HIV testing services.

A review conducted in sub-Saharan Africa found that men were more likely to attend community-based testing venues, such as mobile clinics and workplace services, and more likely to rate these services as acceptable because of their desirable characteristics.^14^ These characteristics included convenience and efficiency when compared with conventional clinic facilities.^10^ The evidence of high acceptability and increased yield of men testing positive for HIV when compared with conventional facilities suggests that community-based HIV services are effective at identifying men in high disease burden communities who have been missed by conventional services.^12^^,^^15^^,^^16^

The “undetectable equals untransmittable” (U=U) message has been implemented worldwide, with strategic emphasis on women and men who have sex with men.^17^ The U=U message is a simple formulation to communicate that persons living with HIV with an undetectable viral load cannot transmit HIV to their sexual partners or to offspring. A recent systematic review found that U=U messaging was significantly associated with improved ART adherence and viral load suppression compared with standard messaging.^18^

In partnership with men from a high disease burden location in Cape Town, South Africa, we used human-centered design workshops to co-create U=U messaging for their peers.^13^ Participants emphasized the benefits of ART and men’s aspirations and identity in their messaging. Following the creation of the messaging, we conducted a randomized trial delivering the U=U messaging versus a standard HIV test invitation to test the effect of the messaging on HIV testing. On intervention days, peer promoters delivered U=U invitations to men in close proximity to a mobile HIV testing clinic. On standard-of-care days, the peer promoters delivered invitations to test for HIV at the mobile clinic using standard messaging. The pilot study showed that men who had received U=U messaging had 60% increased odds of getting tested for HIV compared with those who received a standard invitation.^19^

Considering the evidence above for delivering U=U messaging to men in community-based settings, this study aimed to analyze the impact, cost, and intermediary cost-effectiveness of delivering this intervention on men’s uptake of HIV testing, HIV-positive diagnoses, and linkage to care. The evidence can be used by government policymakers and implementers to help make decisions regarding improving HIV services for men.

## METHODS

### Outcomes

This pilot study had 3 outcomes: impact, cost, and intermediary cost-effectiveness of U=U messaging for improving HIV testing, HIV yield, and linkage to care. The impact of U=U messaging versus standard messaging was based on a randomized trial, which was reported elsewhere^19^ and is summarized in the Results section of this article. Cost and intermediary cost-effectiveness data were sourced from the trial expenditure.

### Message Development

We recruited 40 adult men from the community where the mobile clinic operated. The men attended a 2-day human-centered design workshop where a facilitator presented the evidence for U=U. We asked the participants to design the message so that HIV testing and treatment uptake were framed as a benefit, as opposed to the conventional HIV testing messaging focusing on the dread of an HIV-positive diagnosis. The facilitator guided the men through a series of design activities where men were asked how they would communicate the message to their peers. After the facilitator presented the evidence for U=U, he asked participants to empathize with men and identify aspects of the message that might resonate with their peers. The facilitator asked the men 4 questions to guide their ideation:

1. What might you say to make me confident I will be HIV safe thanks to this pill?
2. What might we do to convince everyone in your community that I’m HIV safe thanks to this pill?
3. How might your favorite brand sell the benefits of this pill to men like you?
4. What might I hear to make me curious to learn more about this pill?

Once participants had identified socially desirable values of the message, the facilitator asked the men to generate a 1-sentence pitch to their peers using these values. After participants generated multiple sentences, they were asked to pitch their sentences to each other and to select their favorite message.

The participants then used the most resonant messages to create a prototype script. The script was initially over 4 minutes long. It was reduced by eliminating repetition and emphasizing the participants’ socially desirable values until it could be delivered in under 40 seconds. The messaging emphasized that individuals who adhere to ART can achieve viral suppression and cannot transmit HIV (U=U):

Hi! My name is (peer promotor’s name), and I work for the Desmond Tutu Centre. Do you know “iMpilo” [“health” in Xhosa, also a play on words because it sounds like “pill”]? iMpilo is the latest *mahala* [“free”] ARV pill that you take once a day if you are infected with HIV. Did you know that iMpilo protects you from getting sick because it reduces HIV in the body—so much so that you can’t infect your partner and family? This is called U=U. It protects you even if you don’t use a condom. Even if you’re drinking. Did you know that? So in no time you’re *Ugrand* [“strong/courageous”] by protecting your partner(s) and family. Your life stays the same and doesn’t change. You and I can show our *kasi* [“community”] how to do this thing one by protecting our kasi *Khusela ikasilam* [“protecting our community”]. Tutu Tester (point to location) can quickly tell you your HIV status and iMpilo for *mahala* [“free”]. Take this invitation with you. See you there!

The intervention sought to reduce the fear associated with HIV testing by highlighting the positive implications of early ART. The message was field-tested with HIV researchers, physicians, and HIV counselors before it was implemented in the study.

### Design and Process

The mobile clinic visited 5 sites in the Klipfontein/Mitchells Plain district in Cape Town, South Africa, and the 5 sites were randomized to intervention or standard-of-care days. We used cluster randomization to investigate the effect of delivering a U=U message to men on HIV testing uptake (primary outcome) and HIV-test positivity (secondary outcome). Computer-generated randomization was used to allocate study group asssignment for each clinic day. Randomization was stratified by location to allocate an equal number of days at each study location.

The intervention consisted of peer promoters delivering the U=U invitation message to adult men (≥18 years) in proximity (≤100 meters) to a mobile clinic conducting HIV testing and screening for common noncommunicable diseases. Peer promoters approached men and delivered the scripted U=U invitation message with a U=U invitation card on intervention days. On control days, the peer promoters delivered a short verbal invitation to visit the mobile clinic for HIV testing and handed men an invitation card. Men who visited the mobile clinic were registered and the registrant conducted informed consent activities.

On each study day, the 2 peer promoters invited around 50 men each (N=100) to test for HIV at the mobile clinic. An a priori calculation estimated that 40 clinic days would result in 4,000 invitations (2,000 per randomization arm) with 80% power to detect a 5% increase in HIV testing uptake, assuming a baseline uptake of 8% (based on a 2-day pilot observation). We used a logistic regression model, which was adjusted for clustering by study day, and included location fixed effects and standard errors. Study enrollment started on March 3, 2020. Due to the COVID-19 lockdown in South Africa, the University of Cape Town closed all non-therapeutic research on March 18, 2020, 12 days after study initiation. The interruption in study days may have introduced some variability, particularly that certain days of the week/locations were overrepresented due to the timing of the lockdown. However, the statistical methods account for such variations, including logistic regression models with location fixed effects and adjusted standard errors for clustering by study day.

### Cost and Cost Comparison

We calculated the mean cost of the U=U intervention and its incremental cost-effectiveness over the control arm. Costs were estimated based on trial expenditure data, analyzed from the provider perspective in 2020 South African rand (ZAR) and converted to US$ using March 2020 average exchange rates (US$1=16.77 ZAR).

Trial costs were sourced from the study expenditure ledger and matched to the trial period of March 2020. Resources shared with other studies were allocated based on input from the project manager and grant administrator. Trial costs were then allocated to each trial arm based on participant volume or days of operation; capital costs, including the creation of the U=U message via human-centered design workshops, were annualized over 8 years for both arms anticipating the life cycle of messaging before requiring a limited redesign. This assumption was informed by the continued use of the message by the study and partner programs in 2026 with minimal modifications, as well as ongoing evaluation of the intervention for HIV testing, linkage-to-care, and viral suppression outcomes. We further assumed that further updates would require iterative updates rather than a complete human-centered design process.

In order to generate costs of use to public-sector planning and budgeting, we created a second scenario in which trial resource use was adjusted for potential implementation in routine public-sector care implemented by the Department of Health. In this scenario, cost was limited to only those items deemed necessary by the team for public-sector implementation, similar to methodology used in previous cost analyses for the South African HIV program based on trial data.^20^^–^^22^ These items included office space, material development including message design costs, and client-facing staff, including peer navigators and counselors conducting the testing. Costs directly associated with research implementation, including staff uniforms, refreshments, tablets, provident and leave, and office overhead support, were excluded from this scenario. The Supplement  details the items included in the trial and routine cost as well as their quantities and cost.

In order to establish the intermediary cost effectiveness of the intervention, we used trial outcome data on the number of men who received an invitation, tested HIV positive, and initiated ART in each arm to calculate the incremental cost of delivering U=U messaging per additional person reached, testing HIV positive, and initiating ART in the U=U arm versus the control arm. Intermediate cost-effectiveness compares the cost to an intermediate program outcome (such as persons testing HIV-positive and persons initiating ART) rather than to process indicators (such as tests done) or final health outcomes (such as infections averted, life-years saved or disability-adjusted life-years averted). Finally, we compared these intermediate cost-effectiveness estimates with estimates for a number of HIV self-test distribution models similarly aimed at increasing HIV testing in men that had been previously established in South Africa, using similar methodology.^22^ In the absence of an established cost-effectiveness threshold for the public sector in South Africa, this allowed us to estimate the relative cost-effectiveness of this intervention in comparison to comparable uses of the country’s HIV testing budget.

### Ethics Statement

This study was approved by the University of Cape Town Human Research Ethics Committee. Written informed consent was obtained from participants.

## RESULTS

Peer promoters delivered 504 U=U invitations over 7 intervention days and 544 standard invitations over 5 control days in March 2020. Of the men who received the standard invitation, 68 (13%) presented for HIV testing, 3 (4%) tested positive for HIV, and 2 (67%) were linked to care (Table 1). Of the men who received a U=U invitation, 112 (22%) presented for HIV testing, 7 (6%) tested positive for HIV, and 3 (43%) were linked to care.^19^

**TABLE 1.** Trial Outcomes of U=U Versus Standard HIV Testing Messaging, Cape Town, South Africa, 2020

	**Intervention Arm** **(U=U Messaging)**	**Control Arm** **(Standard Messaging)**
	**No. (%)** ^a^	**No. (%)** ^a^
Men approached for HIV testing	504	544
Men undergoing HIV testing	112 (22.22)	68 (12.50)
Men testing positive for HIV	7 (6.25)	3 (4.41)
Men initiated on antiretroviral therapy	3 (42.86)	2 (66.67)

^a^ The denominator for each percentage is the No. in the row immediately above the cell.

Abbreviation: U=U, undetectable equals untransmittable.

The average trial cost was $5.40 per participant reached with the U=U message, $388.47 per person testing HIV-positive, and $906.44 per person initiating ART (Table 2). Capital costs including the development costs for the materials made up only 13% of this cost. After adjustment for potential implementation in routine care, these costs were reduced by more than half, to $2.35 per person reached, $168.99 per person testing HIV-positive, and $394.31 per person initiating ART. Capital costs contributed 26% to this cost; without the cost of material development, average cost per participant reached in the routine care scenario was only $1.74.

**TABLE 2.** Total and Average Trial Cost per Outcome and Trial Arm,^a^ Cape Town, South Africa, 2020

	**Intervention Arm** **(U=U Messaging)**			**Control Arm** **(Standard Messaging)**	
	**No. of Participants**	**Trial Cost**	**Routine Cost**	**No. of Participants**	**Trial Cost**
Total cost		$2,719.31	$1,182.92		$3,127.10
Cost per participant approached for HIV testing	504	$5.40	$2.35	544	$6.20
Cost per participant undergoing HIV testing	112	$24.28	$10.56	68	$27.92
Cost per participant testing positive for HIV	7	$388.47	$168.99	3	$446.73
Cost per participant initiated on antiretroviral therapy	3	$906.44	$394.31	2	$1,042.37

^a^ 2020 average exchange rate: US$1=16.77 ZAR.

Abbreviation: U=U, undetectable equals untransmittable.

Within trial costs, the staff, overhead, and research costs were the largest cost items, accounting for 20% to 30% of total costs each (Table 3). After adjustment for routine implementation, the message creation via human-centered design workshops, personnel (peer promoters, testing staff), and supplies costs were the largest cost items.

**TABLE 3.** Distribution of Total and Average Cost of U=U Messaging Into Cost Categories, Cape Town, South Africa, 2020

	**Trial Cost** **(Intervention Arm/U=U Messaging Only)**			**Routine Cost**		
	**Total Cost**	**Average Cost per Participant Approached**	**% of Total**	**Total Cost**	**Average Cost per Client Approached**	**% of Total**
**Capital costs**						
Design	$308.68	$0.61	11%	$307.97	$0.61	26%
Equipment	$32.40	$0.06	1%	–	–	–
Total capital costs	$341.09	$0.68	13%	$307.97	$0.61	26%
**Recurrent costs**						
Staff	$595.60	$1.18	22%	$523.78	$1.04	44%
Supplies	$333.50	$0.66	12%	$243.13	$0.48	21%
Overhead	$758.24	$1.50	28%	$108.04	$0.21	9%
Research	$690.89	$1.37	25%	–	–	–
Total recurrent costs	$2,378.23	$4.72	87%	$874.95	$1.74	74%
**Total**	**$2,719.31**	**$5.40**	**100%**	**$1,182.92**	**$2.35**	**100%**

Abbreviation: U=U, undetectable equals untransmittable.

The cost of U=U messaging per person confirmed HIV-positive and initiating ART was lower than that of conventional HIV testing messaging, based on trial data. It is also at the lower end of cost ranges of HIV self-test distribution models targeted at men established in previous work ($62 to $7,936 per person confirmed positive and $117 to $8,198 per person initiating ART).^22^

## DISCUSSION

Our study investigated the cost and intermediate cost-effectiveness of delivering U=U messaging to improve HIV testing among South African men, based on a trial that found that U=U messaging was effective at improving HIV testing uptake compared with standard HIV testing invitations.

There were 2 main findings related to U=U messaging. First, the cost analysis demonstrated that U=U messaging was comparable to other efforts to increase uptake among men, a high priority population for reaching the UNAIDS 95-95-95 targets. Second, the cost of delivering U=U messaging was lower than conventional messaging per person confirmed HIV-positive as well as per person subsequently initiating ART. These findings show that delivering tailored U=U messaging can increase HIV testing uptake and subsequent ART initiation while lowering costs per person reached when compared with untargeted, passive case-finding methods, such as providing HIV self-testing distribution models.

### Limitations

The strength of our findings stand beside a number of limitations to our analysis. Firstly, we based our cost analysis on expenditure data instead of a bottom-up estimation of resource use and cost, which means we could have over- or underestimated the true cost of the Intervention. Related to this, the study was discontinued early due to the COVID-19 lockdown with only 12 days and 1,048 of a projected 4,000 invitations delivered. While there was sufficient power for the primary outcome, a larger sample size may have had more robust insights. Secondly, even though we adjusted our cost for potential routine care implementation by including those cost items that were relevant only to the trial, we did not further adjust for lower quantities or public sector prices/salaries, so our routine cost scenario might still be an overestimate. Lastly, because the study focused on short-term outcomes related to HIV testing uptake and the impact of U=U messaging on linkage to care, overall cost-effectiveness over extended periods remains an area for future research. For example, in addition to HIV testing uptake and ART initiation, U=U messaging may have the potential to improve ART adherence and retention, which were not explored in this study. Despite these limitations, this study offered valuable insights into the potential of U=U messaging, emphasizing the need for further research to optimize strategies for sustainable and cost-effective HIV prevention efforts in similar high HIV burden settings.

## CONCLUSIONS

As South Africa approaches the UNAIDS 95-95-95 targets,^9^ effective and cost-effective tools that can increase the yield of hard-to-reach populations are required. Delivering tailored U=U messaging can increase HIV testing and ART uptake among men^19^^,^^23^ while reducing average costs over standard untargeted HIV testing messaging and other HIV self-test distribution models. Since this was a pilot trial, larger studies investigating the effect of U=U on HIV testing uptake, ART initiation, treatment adherence, and viral load suppression are required for generalizability. Along with other, non-conventional strategies,^22^^,^^24^ U=U messaging may be important for improving HIV outcomes for men and their partners and achieving epidemic control.

## Notes

**Peer Reviewed**
